# New Remedies

**Published:** 1885-02

**Authors:** 


NEW REMEDIES.

Professor H. Senator, in a lecture delivered Nov. 20, 1884, before the Society of the Charité Physicians (*Berliner Klinische Wochenschrift*, Jan. 5, 1885), has expressed his views as to the therapeutic value of certain exotic remedies, which are employed, to a greater or less extent, by American physicians.

He has tried, on an extensive scale, the tincture of *cascara sagrada*, as prepared by Messrs. Parke, Davis & Co., and expresses great satisfaction with the results. In physiological action it deserves a place between rhubarb and senna. It has the advantage over both of these remedies of producing an effect in smaller dose.

*Euonymin*, derived from *evonymus atropurpureus*, is referred to the list of cholagogue cathartics, allied to podophyllin.

He ascribes decided hypnotic qualities to the extract of *piscidia erythrina.* It is a more reliable remedy than paraldehyde or cannabin. The remedy is especially useful in migraine. It does not, however, produce so sound sleep as chloral or opium. *Muriate of cocaine* has produced, in Dr. Senator's hands, transitory anæsthesia of the mucous membrane of the rectum, urethra and bladder, in a variety of pathological conditions. In one case of *tabes dorsalis*, cocaine suppositories relieved the most violent, boring pains in the rectum, to which Westphal has recently called attention.

*Oil of wintergreen* possesses few advantages over salicylic acid in the treatment of articular rheumatism. It may be exhibited when the patient is averse to salicylic acid. When given in doses sufficient to control disease, it is productive of all the disagreeable symptoms of salicylic acid.

*Picrotoxin* was exhibited to control night sweats in forty cases of tuberculosis. In two-thirds of all the cases, the desired effect was observed.

*Agaricin* is also an effective remedy in the night sweats of phthisis. In large doses, however, it causes diarrhœa. It is also very expensive.

Both picrotoxin and agaricin are comparable in their action to atropine.

In two cases of *tænia mediocanellata*, *pelletierine* proved effective. In one case the worm and head were expelled without doubt, in the other case, with a high degree of probability. The cost of the remedy is so great that *filix mas* will not be immediately superseded.

